# Supplementary material for: Single cell landscape of sex differences in the different courses of multiple sclerosis
Source: Biol Sex Differ. 2026 Jul 24;17:170. doi: 10.1186/s13293-026-00957-y (PMC13693195; doi:10.1186/s13293-026-00957-y)
Supplement: Supplementary file 1 [file 13293_2026_957_MOESM1_ESM.docx]

**TABLE OF CONTENTS**

**Supplementary Notes**

**Supplementary Note**

Description of the analyzed datasets.	***3***

**Supplementary Tables**

Table S1. Description of the selected datasets.	***4***

Table S2. Sex differential activated effectors in secondary progressive multiple sclerosis.	***8***

Table S3. General classification of female enriched functions from cluster 4 of the astrocyte-microglia-neuron triad in secondary progressive multiple sclerosis (main manuscript Fig. 2A).	***9***

Table S4. General classification of male enriched functions from cluster 1 of the astrocyte-microglia-neuron triad in secondary progressive multiple sclerosis (main manuscript figure 2B).	***12***

Table S5. Sex differentially expressed genes in oligodendrocytes and OPCs.	***13***

Table S6. Human leukocyte antigens dysregulated in multiple sclerosis.	***13***

Table S7. List of R packages and versions implemented in the bioinformatic workflow.	***15***

Table S8. Signalling pathways analysed based on the type of sample examined: nervous tissue (USCS-MS dataset) and blood (GSE144744 cohorts).	***15***

**Supplementary Figures**

Figure S1. Results of data screening process.	***16***

Figure S2. UCSC-MS dataset preprocessing.	***20***

Figure S3. GSE144744 cohort 1 preprocessing.	***22***

Figure S4. GSE144744 cohort 3 preprocessing.	***23***

Figure S5. Summary of significant sex differences in secondary progressive multiple sclerosis.	***23***

Figure S6. Associations between number of cells and number of differentially expressed genes across cell types and comparisons for secondary progressive multiple sclerosis dataset. ***23***

Figure S7. Astrocyte sex-differential atlas in secondary progressive multiple sclerosis.	***25***

Figure S8. Microglia sex-differential atlas in secondary progressive multiple sclerosis.	***27***

Figure S9. Neurons sex-differential atlas in secondary progressive multiple sclerosis.	***29***

Figure S10. Oligodendrocytes sex-differential atlas in secondary progressive multiple sclerosis.	***30***

Figure S11. Oligodendrocyte precursor cells sex-differential atlas in secondary progressive multiple sclerosis.	***31***

Figure S12. Intersection of significant results among brain cell types for secondary progressive multiple sclerosis.	***32***

Figure S13. Significant results in the analyses of (A) signaling pathway effector activation and (B) cell-cell communication for the secondary progressive form of multiple sclerosis..	***33***

Figure S14. Upset plot depicting the overlap of sex-differentially expressed synaptic genes across neuronal subtypes. ***34***

Figure S15. Sex-differential patterns in glutamatergic and GABAergic signaling pathways in the validation dataset.	***35***

Figure S16. Sex differential signaling pathways of (A) cholinergic, (B) serotonergic and (C) dopaminergic synapses in neurons from secondary progressive multiple sclerosis.	***37***

Figure S17. Significant functions in oligodendrocytes and oligodendrocyte precursor cells (OPCs) in secondary progressive multiple sclerosis.	***37***

Figure S18. Significant sex differences summary in relapsing-remitting multiple sclerosis.	***39***

Figure S19. Associations between number of cells and number of differentially expressed genes across cell types and comparisons for relapsing-remitting multiple sclerosis dataset	***39***

Figure S20. CD4+ T cells sex-differential atlas in relapsing-remitting multiple sclerosis.	***41***

Figure S21. CD8+ T cells sex-differential atlas in relapsing-remitting multiple sclerosis.	***43***

Figure S22. Monocytes sex-differential atlas in relapsing-remitting multiple sclerosis.	***44***

Figure S23. Monocytes sex-differential atlas in relapsing-remitting multiple sclerosis.	***46***

Figure S24. (A) B cells and (B) dendritic cells sex-differential atlas in relapsing-remitting multiple sclerosis.	***47***

Figure S25. Detailed sex-differential significant changes in the activation of signaling pathways and interaction strengths in cell-cell communication for relapsing-remitting multiple sclerosis	***49***

Figure S26. Upset plot for genes more expressed in males than females by cell type in relapsing-remitting multiple sclerosis. ***49***

Figure S27. Summary of sex differences summary in primary progressive multiple sclerosis...*……………………………………...…………………………………………………....****51***

Figure S28. Associations between number of cells and number of differentially expressed genes across cell types and comparisons for primary progressive multiple sclerosis	dataset	***51***

Figure S29. CD4+ T cells sex-differential atlas in primary progressive multiple sclerosis.	***53***

Figure S30. CD8+ T cells sex-differential atlas in primary progressive multiple sclerosis.	***55***

Figure S31. NK cells sex-differential atlas in primary progressive multiple sclerosis.	***57***

Figure S32. Monocytes sex-differential atlas in primary progressive multiple sclerosis.	***59***

Figure S33. (A, C) B cells and (B, D) dendritic cells sex-differential atlas in primary progressive multiple sclerosis.	***61***

Figure S34. Detailed sex-differential significant changes in the activation of signaling pathways for primary progressive multiple sclerosis.	***62***

Figure S35. Detailed sex-differential significant changes in the cell-cell communication interaction strengths for primary progressive multiple sclerosis.	***63***

Figure S36. Protein-protein interaction network of the genes that provide clustering of RRMS and PPMS immune system cell types according to their differential sex profile……………………………………………………………………………………….…….…..***64***

Figure S37.Cell distribution by sample (donor)......................................................................	***65***

**Supplementary Note**

**Description of the analyzed datasets**

UCSC-MS

This study included postmortem brain tissue obtained from the UK Multiple Sclerosis Tissue Bank and the NIH NeuroBioBank, including a total of 12 multiple secondary progressive multiple sclerosis (SPMS) and 9 control samples processed for single-nucleus RNA sequencing (snRNA-seq). Samples comprised cortical gray matter and adjacent subcortical white matter. The MS cases represent the disease course without exposure to immunomodulatory treatments. Control variables such as age, sex, postmortem interval, and RNA integrity screening did not differ significantly between MS and control groups.

GSE144744 cohorts

The study GSE144744 included peripheral blood mononuclear cells (PBMCs). Samples were retrieved from the Biobank of the Institute of Neuroimmunology and Multiple Sclerosis (Germany). It comprises a heterogeneous set of samples divided into three independent MS patient cohorts, together with matched healthy controls for the specific characteristics of each cohort. Unfortunately, cohort 2 composed by untreated RRMS samples was excluded from our analysis, as it only contains female samples. The remaining two cohorts were incorporated into our analyses.

Cohort 1 consisted of nine RRMS patients who were sampled longitudinally, both before and after treatment with the integrin-blocking antibody natalizumab. MS patients presented active lesions detected by contrast-enhanced magnetic resonance imaging, although data were missing for two samples. Since our aim was to characterize the natural state of immunity in MS, we excluded the treated samples to avoid therapy-related effects that could strongly bias the results.

Meanwhile, cohort 3 from the GSE144744 study consisted of PBMCs samples from nine patients diagnosed with primary progressive multiple sclerosis (PPMS), all of them untreated at the time of sampling.

Macnair et. al. 2025 (validation dataset)

This study included postmortem brain tissue samples, comprising 156 snap-frozen brain tissue blocks obtained at autopsy from 54 MS patients and 26 control donors. Samples included cortical gray matter and white matter from frontal, parietal, and temporal regions. They were collected through the Netherlands Brain Bank, the UK MS Tissue Bank, and the Edinburgh Brain Bank. MS and control groups were matched for age and sex. For the validation of our work, and to reproduce the characteristics of the UCSC-MS dataset, we analyzed the SPMS gray matter samples.

|  | **UCSC-MS** | **GSE144744 cohort 1** | **GSE144744 cohort 3** | **Validation dataset **** |
| --- | --- | --- | --- | --- |
| **Database** | UCSC Cell Browser | GEO | GEO | Zenodo |
| **Sequencing type** | snRNA-seq | scRNA-seq | scRNA-seq | snRNA-seq |
| **Sequencing method** | 10x Genomics | 10x Genomics | 10x Genomics | 10x Genomics |
| **Sequencing platform** | Illumina HiSeq 2500 | Illumina Nextseq 500 | Illumina Nextseq 500 | Illumina NovaSeq 6000 |
| **Sample type** | *Post-mortem* brain tissue | PBMCs | PBMCs | *Post-mortem* brain tissue |
| **MS subtype** | SPMS | RRMS | PPMS | SPMS |
| **Patients by condition *** | 4:8:5:4 | 5:4:5:5 | 3:3:6:6 | 7:16:8:13 |
| **Status of the accessible data** | unfiltered raw counts | filtered raw counts | filtered raw counts | filtered raw counts |
| **Cell count** | 48,919 | 71,592 | 265,342 | 120,904 |
| **Gene count** | 65,217 | 15,354 | 15,354 | 33,939 |
| **PMID** | 31316211 | 33748804 | 33748804 | 39708806 |

**Table S1. Description of the selected datasets.** *Female control:female case:male control:male case. **Number of samples, cells, and genes analyzed for the subset under study (neurons from the gray matter of SPMS samples). *MS: multiple sclerosis; PBMCs: peripheral blood mononuclear cells; PMID: PubMed ID; PPMS: primary progressive MS; SPMS: secondary progressive MS; RRMS: relapsing-remitting MS.*

| **Cell type** | **Pathway** | **Effectors more activated in females** | **Effectors more activated in males** |
| --- | --- | --- | --- |
| Astrocytes | Adherens junction | CTNND1** |  |
|  | Amphetamine addiction | GRIA1 |  |
|  | AMPK signaling pathway | ACACA, ACACB |  |
|  | Amyotrophic lateral sclerosis (ALS) | SLC1A2 |  |
|  | Apoptosis |  | TUBA1B |
|  | Axon guidance | FYN |  |
|  | cAMP signaling pathway | PTCH1 |  |
|  | cGMP-PKG signaling pathway | PDE3A |  |
|  | Epstein-Barr virus infection |  | HLA-A |
|  | Glutamatergic synapse | ADRBK1, GRIA1,  GRM3 |  |
|  | Hedgehog signaling pathway | GLI1* |  |
|  | Retrograde endocannabinoid signaling | GRIA1 |  |
|  | Taste transduction | GABBR1 |  |
| Oligodendrocytes | Tight junction |  | CTNNA1 ACTB ACTN4 |
| Neurons | Adherens junction |  | ACTB, ACTB ACTN4 CTNNA1, CTNND1, CTNND1**, NLK, RHOA |
|  | Alzheimer's disease |  | ATF6, GAPDH, MAPT |
|  | Amphetamine addiction | GRIA1, GRIN3A, GRIN3A* | MAOA, MAOA* |
|  | AMPK signaling pathway | ACACA, CFTR, FOXO1, GYS1 | EEF2, PFKL |
|  | Amyotrophic lateral sclerosis (ALS) |  | DERL1, H2O, PPP3CA, RAB5A, SLC1A2, TOMM40 |
|  | Apoptosis |  | MCL1, TUBA1B |
|  | Axon guidance | FYN, RASA1, UNC5D DCC | CDC42*, CFL1, MAPK1*, PAK4, RAC1****, RHOA***, ROCK1, ROCK1*, UNC5D PTPN11 |
|  | Calcium signaling pathway | 3,5-Cyclic AMP, Sodium cation, Sodium cation* | ADCY1, MYLK4, PDE1A, Sphingosine 1-phosphate |
|  | cAMP signaling pathway |  | BAD, JUN, PAK1 |
|  | Cell cycle |  | RB1* |
|  | cGMP-PKG signaling pathway | 3,5-Cyclic AMP, GTF2I | ATP1B4, ATP2B1, BAD, CREB3, ITPR1, PDE2A, PPIF, SLC8A2 |
|  | Chemokine signaling pathway | PRKACA | PAK1 |
|  | Cholinergic synapse | CACNA1A, CHRM1*, ITPR1, PRKCA | BCL2, CHRNA7, CREB3, FOS, KCNJ3 |
|  | Circadian rhythm |  | BHLHE41, CRY1 |
|  | Dopaminergic synapse | CACNA1A | CACNA1C, CAMK2A, CREB3, FOS, GRIN2A, KCNJ3, KIF5A GRIA1 |
|  | Epstein-Barr virus infection | GSK3B | HLA-A |
|  | ErbB signaling pathway | GSK3B | BAD, CDKN1B |
|  | Focal adhesion |  | BAD, BIRC2, CCND1, ZYX ACTB |
|  | FoxO signaling pathway |  | CDKN2D, PLK4 |
|  | GABAergic synapse | ADCY1, CACNA1A, CACNA1A*, GABRA1 GPHN, PRKACA | KCNJ6 |
|  | Glucagon signaling pathway |  | PFKL |
|  | Glutamatergic synapse | GRIA1, PRKACA* | ADRBK1, GRIN3A DLG4, GRM3, ITPR1 HOMER3 HOMER3, MAPK1, PRKACA, TRPC1 |
|  | Hedgehog signaling pathway |  | CCND1, SMO |
|  | HIF-1 signaling pathway |  | PDHA1 |
|  | Hippo signaling pathway |  | SAV1 |
|  | Huntington's disease |  | DLG4 HTT |
|  | Inflammatory mediator regulation of TRP channels | ASIC2, TRPV1, TRPV1** |  |
|  | Long-term potentiation |  | ITPR1 |
|  | Longevity regulating pathway - mammal | NFKB1 | RPS6KB1, SOD2 |
|  | MAPK signaling pathway |  | MAP3K2, MAP3K4 |
|  | Morphine addiction | ADCY1, CACNA1A, GABRR3, GABRR3*, GABRR3**, PRKACA | 3,5-Cyclic AMP*, KCNJ3, KCNJ6, KCNJ6*, KCNJ6** |
|  | Olfactory transduction |  | PDE1A |
|  | PI3K-Akt signaling pathway |  | PKN3, C8orf44-SGK3 |
|  | PPAR signaling pathway |  | UBC |
|  | Ras signaling pathway |  | ELK1, MAPK8, MLLT4, MLLT4*, PAK4, PIK3R5*, RAF1*, RALGDS*, RAPGEF5*, RHOA, RIN1*, TIAM1* |
|  | Regulation of actin cytoskeleton |  | CFL1 ACTB |
|  | Retrograde endocannabinoid signaling | ADCY1, CACNA1A, CACNA1A*, GRIA1, ITPR1, PRKACA, PRKCA |  |
|  | Serotonergic synapse | CACNA1A, PRKCA, TRPC1 | CACNA1C, KCNJ3, PLA2G4B |
|  | Sphingolipid signaling pathway |  | SMPD1 |
|  | Taste transduction | GABBR1, GABRA1 | PLCB2 |
|  | TGF-beta signaling pathway |  | SMAD4*** |
|  | Tight junction | AKT3, IGSF5 IGSF5 | ACTB MLLT4, ACTB MYL12B, ACTB SHROOM1, CTNNA1 ACTB ACTN4, IGSF5 MAGI1, IGSF5 MPDZ |
|  | TNF signaling pathway |  | DNM1L, JUN |
|  | VEGF signaling pathway |  | BAD |
|  | Wnt signaling pathway |  | MAPK8, ROCK2 |

#### **Table S2. Sex differential activated effectors in secondary progressive multiple sclerosis.** * Indicates different proteins from the same complex.

| **GO ID parent** | **Description parent term** | **GO ID** | **Description** |
| --- | --- | --- | --- |
| GO:0006491 | N-glycan processing | GO:0006491 | N-glycan processing |
| GO:1902430 | negative regulation of amyloid-beta formation | GO:1902430 | negative regulation of amyloid-beta formation |
|  |  | GO:0034205 | amyloid-beta formation |
| GO:0045892 | negative regulation of transcription, DNA-templated | GO:0045892 | negative regulation of transcription, DNA-templated |
|  |  | GO:0000381 | regulation of alternative mRNA splicing, via spliceosome |
|  |  | GO:0048024 | regulation of mRNA splicing, via spliceosome |
| GO:0006511 | ubiquitin-dependent protein catabolic process | GO:0006511 | ubiquitin-dependent protein catabolic process |
|  |  | GO:0018105 | peptidyl-serine phosphorylation |
|  |  | GO:0000209 | protein polyubiquitination |
|  |  | GO:0046777 | protein autophosphorylation |
|  |  | GO:0035335 | peptidyl-tyrosine dephosphorylation |
|  |  | GO:0018107 | peptidyl-threonine phosphorylation |
|  |  | GO:0070936 | protein K48-linked ubiquitination |
|  |  | GO:0018401 | peptidyl-proline hydroxylation to 4-hydroxy-L-proline |
|  |  | GO:0033135 | regulation of peptidyl-serine phosphorylation |

#### **Table S3. General classification of female enriched functions from cluster 4 of the astrocyte-microglia-neuron triad in secondary progressive multiple sclerosis (main manuscript Fig. 2A).** Classification performed with the *rrvgo* R package setting medium threshold (value=0.7). *GO: Gene Ontology.*

| **GO ID parent term** | **Description parent term** | **GO ID** | **Description** |
| --- | --- | --- | --- |
| GO:0006198 | cAMP catabolic process | GO:0006198 | cAMP catabolic process |
|  |  | GO:0019673 | GDP-mannose metabolic process |
| GO:0002181 | cytoplasmic translation | GO:0002181 | cytoplasmic translation |
|  |  | GO:0045727 | positive regulation of translation |
|  |  | GO:0006446 | regulation of translational initiation |
|  |  | GO:1990440 | positive regulation of transcription from RNA polymerase II promoter in response to endoplasmic reticulum stress |
| GO:0043968 | histone H2A acetylation | GO:0043968 | histone H2A acetylation |
|  |  | GO:0000338 | protein deneddylation |
| GO:0006120 | mitochondrial electron transport, NADH to ubiquinone | GO:0006120 | mitochondrial electron transport, NADH to ubiquinone |
|  |  | GO:0006096 | glycolytic process |
|  |  | GO:0006123 | mitochondrial electron transport, cytochrome c to oxygen |
|  |  | GO:0006119 | oxidative phosphorylation |
|  |  | GO:0006122 | mitochondrial electron transport, ubiquinol to cytochrome c |
| GO:0000398 | mRNA splicing, via spliceosome | GO:0000398 | mRNA splicing, via spliceosome |
|  |  | GO:0000381 | regulation of alternative mRNA splicing, via spliceosome |
|  |  | GO:0048026 | positive regulation of mRNA splicing, via spliceosome |
|  |  | GO:0048025 | negative regulation of mRNA splicing, via spliceosome |
|  |  | GO:0000956 | nuclear-transcribed mRNA catabolic process |
| GO:0043154 | negative regulation of cysteine-type endopeptidase activity involved in apoptotic process | GO:0043154 | negative regulation of cysteine-type endopeptidase activity involved in apoptotic process |
|  |  | GO:0031397 | negative regulation of protein ubiquitination |
|  |  | GO:0032148 | activation of protein kinase B activity |
|  |  | GO:0045737 | positive regulation of cyclin-dependent protein serine/threonine kinase activity |
|  |  | GO:0031954 | positive regulation of protein autophosphorylation |
|  |  | GO:0031396 | regulation of protein ubiquitination |
|  |  | GO:0032515 | negative regulation of phosphoprotein phosphatase activity |
|  |  | GO:1905907 | negative regulation of amyloid fibril formation |
|  |  | GO:1904667 | negative regulation of ubiquitin protein ligase activity |
|  |  | GO:1902949 | positive regulation of tau-protein kinase activity |
| GO:0010894 | negative regulation of steroid biosynthetic process | GO:0010894 | negative regulation of steroid biosynthetic process |
|  |  | GO:0150172 | negative regulation of steroid biosynthetic process |
| GO:0051092 | positive regulation of NF-kappaB transcription factor activity | GO:0051092 | positive regulation of NF-kappaB transcription factor activity |
|  |  | GO:0032092 | positive regulation of protein binding |
|  |  | GO:0043392 | negative regulation of DNA binding |
| GO:0008216 | spermidine metabolic process | GO:0008216 | spermidine metabolic process |
| GO:0006511 | ubiquitin-dependent protein catabolic process | GO:0006511 | ubiquitin-dependent protein catabolic process |
|  |  | GO:0043161 | proteasome-mediated ubiquitin-dependent protein catabolic process |
|  |  | GO:0030433 | ubiquitin-dependent ERAD pathway |
|  |  | GO:0032436 | positive regulation of proteasomal ubiquitin-dependent protein catabolic process |
|  |  | GO:0016241 | regulation of macroautophagy |
|  |  | GO:0032435 | negative regulation of proteasomal ubiquitin-dependent protein catabolic process |
|  |  | GO:0061136 | regulation of proteasomal protein catabolic process |
|  |  | GO:0019941 | modification-dependent protein catabolic process |

#### **Table S4. General classification of male enriched functions from cluster 1 of the astrocyte-microglia-neuron triad in secondary progressive multiple sclerosis (main manuscript Fig. 2B).** Classficacion performed with the *rrvgo* R package setting medium threshold (value=0.7). GO: Gene Ontology.

| **Oligodendrocytes** | |
| --- | --- |
| Increased in females | AMER2, RASGEF1B, SLC26A3, TMEM144, ANK3, ANO4, CADM2, CLDN11, CTNND2, DYSF, FCHSD2, GPRC5B, IL1RAPL1, L3MBTL4, MALAT1, MARCH1, NCAM2, NLGN1, OLMALINC, PLXDC2, SPOCK1, LINC00685, LPHN3 |
| Increased in males | BCYRN1, CAMK2B, CCK, CHN1, DPYSL5, ENC1, MAP1B, MIR219A2, NRGN, TUBB2B, AATK, BCAS1, CERCAM, MGAT5, MIR325HG, PDE4D, RBFOX1, SLC5A11 |
| **OPCs** | |
| Increased in females | HIF3A, GPNMB, KAZN, PARD3, QKI, SMOC1, TNR, VCAN |
| Increased in males | MAP1B, NRGN, SYT1 |

#### **Table S5. Sex differentially expressed genes in oligodendrocytes and OPCs.** Listed genes are significant in all 3 comparisons: IDF: impact of disease in females (MS females vs control females); IDM: impact of disease in males (MS males vs control males); SDID: sex differential impact of disease ((MS females vs control females) - (MS males vs control males)). OPCs: oligodendrocyte precursor cells.

|  | **RRMS** | | **PPMS** | |
| --- | --- | --- | --- | --- |
|  | **Females** | **Males** | **Females** | **Males** |
| CD4+ T cells |  |  |  |  |
| CD8+ T cells |  |  |  | HLA-DPA1, HLA-DPB1 |
| NK cells |  |  |  | HLA-DPA1, HLA-DPB1 |
| Monocytes | HLA-DQB1, HLA-DQA1 | HLA-DRB5 |  | HLA-A, HLA-DRA, HLA-DRB1,  HLA-DPB1 |

#### **Table S6. Human leukocyte antigens dysregulated in multiple sclerosis.** ‘HLA-’ genes have been filtered out among those significant in all 3 comparisons: IDF: impact of disease in females (MS females vs control females); IDM: impact of disease in males (MS males vs control males); SDID: sex differential impact of disease ((MS females vs control females) - (MS males vs control males)). RRMS: relapsing-remitting multiple sclerosis; PPMS: primary progressive multiple sclerosis; NK: natural killer.

| **Package** | **Version** |
| --- | --- |
| SingleCellExperiment | 1.16.0 |
| scuttle | 1.4.0 |
| scDblFinder | 1.8.0 |
| scran | 1.22.1 |
| scater | 1.22.0 |
| PCAtools | 2.6.0 |
| igraph | 1.2.11 |
| bluster | 1.4.0 |
| BRETIGEA | 1.0.3 |
| SingleR | 1.8.1 |
| celldex | 1.4.0 |
| MAST | 1.20.0 |
| STRINGdb | 2.6.5 |
| topGO | 2.46.0 |
| org.Hs.eg.db | 3.14.0 |
| hipathia | 2.10.0 |
| ggplot2 | 3.4.0 |
| dplyr | 1.0.8 |
| Matrix | 1.5.1 |
| BiocParallel | 1.28.3 |
| pheatmap | 1.0.12 |
| reshape2 | 1.4.4 |
| ggpubr | 0.4.0 |
| simplifyEnrichment | 1.4.0 |
| gridExtra | 2.3.0 |
| ComplexHeatmap | 2.13.4 |
| circlize | 0.4.14 |
| CellChat | 1.6.1 |
| rrvgo | 1.6.0 |

#### **Table S7.** List of R packages and versions implemented in the bioinformatic workflow.

| **Sample type** | **KEGG ID pathways** |
| --- | --- |
| Nervous tissue | hsa03320, hsa04010, hsa04012, hsa04014, hsa04015, hsa04020, hsa04022, hsa04024, hsa04062, hsa04064, hsa04066, hsa04068, hsa04071, hsa04072, hsa04110, hsa04115, hsa04150, hsa04151, hsa04152, hsa04210, hsa04211, hsa04218, hsa04310, hsa04330, hsa04340, hsa04350, hsa04360, hsa04370, hsa04390, hsa04510, hsa04520, hsa04530, hsa04540, hsa04612, hsa04620, hsa04621, hsa04622, hsa04623, hsa04630, hsa04668, hsa04710, hsa04713, hsa04720, hsa04722, hsa04723, hsa04724, hsa04725, hsa04726, hsa04727, hsa04728, hsa04730, hsa04740, hsa04742, hsa04750, hsa04810, hsa04915, hsa04917, hsa04921, hsa04922, hsa05010, hsa05012, hsa05014, hsa05016, hsa05020, hsa05030, hsa05031, hsa05032, hsa05169 |
| Blood | hsa03320, hsa04010, hsa04012, hsa04014, hsa04015, hsa04020, hsa04022, hsa04024, hsa04062, hsa04064, hsa04066, hsa04068, hsa04071, hsa04072, hsa04115, hsa04150, hsa04151, hsa04152, hsa04210, hsa04211, hsa04218, hsa04310, hsa04330, hsa04340, hsa04350, hsa04370, hsa04390, hsa04510, hsa04520, hsa04530, hsa04540, hsa04610, hsa04612, hsa04620, hsa04621, hsa04622, hsa04623, hsa04630, hsa04650, hsa04660, hsa04662, hsa04666, hsa04668, hsa04670, hsa04710, hsa04713, hsa04750, hsa04810, hsa04915, hsa04917, hsa05169 |

#### **Table S8. Signalling pathways analysed based on the type of sample examined: nervous tissue (USCS-MS dataset) and blood (GSE144744 cohorts).** *KEGG: Kyoto Encyclopedia of Genes and Genomes; ID: identifier.*

**Figure S1. Results of data screening process.** Specification of remaining study number (n) through the identification, screening, eligibility, and inclusion phases, along with the corresponding exclusion justifications.

#### **Figure S2. UCSC-MS dataset preprocessing.** (A) Violin plots before and after quality control filtering. Representation of the mitochondrial gene ratio (Y-axis) for each sample (X-axis). Each dot represents a cell, coloured according to the condition and sex of the subject. (B) Number of expressed genes (Y-axis) versus the library size (X-axis) for each sample before (top) and after (bottom) the quality control filtering step. Each dot represents a cell, coloured according to the sample identification. (C) Boxplots of the sample library size before and after normalization. (D) Percentage of explained variability for each principal component. Horizontal bar marks the number of principal components selected for further dimensionality reduction analyses. PC: Principal Component. (E) Cell distribution by group. Each dot represents a cell, separated and coloured by the condition and sex of the subjects. (F) tSNE embedding where each dot represents a cell type, coloured according to the assigned cluster which are also denoted by numbers. t-Distributed Stochastic Neighbour Embedding (G) Percentage of variance explained by each covariate reported in the original dataset (Y-axis) for each principal component (X-axis).

#### **Figure S3. GSE144744 cohort 1 preprocessing.** (A) Violin after quality control filtering from the original authors. Representation of the mitochondrial gene ratio (Y-axis) for each sample (X-axis). Each dot represents a cell, coloured according to the condition and sex of the subject. (B) Number of expressed genes (Y-axis) versus the library size (X-axis) for each sample after the quality control filtering step. Each dot represents a cell, coloured according to the sample identification. (C) Boxplots of the sample library size before and after normalization. (D) Percentage of explained variability for each principal component. Horizontal bar marks the number of principal components selected for further dimensionality reduction analyses. PC: Principal Component. (E) Cell distribution by group. Each dot represents a cell, separated and coloured by the condition and sex of the subjects. (F) UMAP embedding where each dot represents a cell type, coloured according to the assigned cluster which are also denoted by numbers. t-Distributed Stochastic Neighbour Embedding (G) Percentage of variance explained by each covariate reported in the original dataset (Y-axis) for each principal component (X-axis). Each value reflects the proportion of variance of a given PC explained by a single covariate without accounting for other covariates or PCs.

#### **Figure S4. GSE144744 cohort 3 dataset preprocessing.** (A) Violin after quality control filtering from the original authors. Representation of the mitochondrial gene ratio (Y-axis) for each sample (X-axis). Each dot represents a cell, coloured according to the condition and sex of the subject. (B) Number of expressed genes (Y-axis) versus the library size (X-axis) for each sample after the quality control filtering step. Each dot represents a cell, coloured according to the sample identification. (C) Boxplots of the sample library size before and after normalization. (D) Percentage of explained variability for each principal component. Horizontal bar marks the number of principal components selected for further dimensionality reduction analyses. PC: Principal Component. (E) Cell distribution by group. Each dot represents a cell, separated and coloured by the condition and sex of the subjects. (F) UMAP embedding where each dot represents a cell type, coloured according to the assigned cluster which are also denoted by numbers. t-Distributed Stochastic Neighbour Embedding (G) Percentage of variance explained by each covariate reported in the original dataset (Y-axis) for each principal component (X-axis).

#### **Figure S5. Summary of significant sex differences in secondary progressive multiple sclerosis.** (A) Expression pattern of marker genes (Y-axis) in astrocytes, microglia, neurons, oligodendrocytes and oligodendrocyte precursor cells (OPCs) (Y-axis). Each dot represents a cell, coloured according to the cell type noted. Marker genes have been selected from bibliographic research. (B) Number of cells identified for each cell type (X-axis) based on condition (Y-axis) and sex (colour). MS: multiple sclerosis; OPCs: oligodendrocyte precursor cells.

**Figure S6. Associations between number of cells and number of differentially expressed genes across cell types and comparisons for secondary progressive multiple sclerosis dataset.** Each point represents a specific cell type and comparison, colored by comparison group and shaped by cell type. The x-axis indicates the number of cells per condition, while the y-axis shows the number of significant differentially expressed genes (FDR < 0.05 and |logFC| > 0.5). Linear regression lines with 95% confidence intervals are shown in grey. Spearman correlation coefficients (rho) are indicated for each analysis.

#### **Figure S7. Astrocyte sex-differential atlas in secondary progressive multiple sclerosis.** (A) Volcano plots of differential gene expression results. Each point represents a gene distributed by its magnitude of change (logFC, X-axis) and its significance (-log10(p.fitted value), X-axis) for each comparison. IDF: impact of disease in females (MS females vs control females); IDM: impact of disease in males (MS males vs control males); SDID: sex differential impact of disease ((MS females vs control females) - (MS males vs control males)). Protein-protein interaction network of genes increased in females (B, orange) and males (C, green) from those significant in all three comparisons. Edge thickness is directly proportional to the structural and functional confidence of the interaction. PPI: protein-protein interaction. (D) Degree of similarity between sex-differential enriched functions in females (left horizontal orange bars) and males (left horizontal green bars). Each row and column corresponds with a significant function. Blue intensity indicates the degree of similarity. Clusters are shown at the bottom, with the associated word cloud on the right of the plot.

#### **Figure S8. Microglia sex-differential atlas in secondary progressive multiple sclerosis.** (A) Volcano plots of differential gene expression results. Each point represents a gene distributed by its magnitude of change (logFC, X-axis) and its significance (-log10(p.fitted value), X-axis) for each comparison. IDF: impact of disease in females (MS females vs control females); IDM: impact of disease in males (MS males vs control males); SDID: sex differential impact of disease ((MS females vs control females) - (MS males vs control males)). Protein-protein interaction network of genes increased in females (B, orange) and males (C, green) from those significant in all three comparisons. Edge thickness is directly proportional to the structural and functional confidence of the interaction. PPI: protein-protein interaction. (D) Degree of similarity between sex-differential enriched functions in females (left horizontal orange bars) and males (left horizontal green bars). Each row and column corresponds with a significant function. Blue intensity indicates the degree of similarity. Clusters are shown at the bottom, with the associated word cloud on the right of the plot.

#### **Figure S9. Neurons sex-differential atlas in secondary progressive multiple sclerosis.** (A) Volcano plots of differential gene expression results. Each point represents a gene distributed by its magnitude of change (logFC, X-axis) and its significance (-log10(p.fitted value), X-axis) for each comparison. IDF: impact of disease in females (MS females vs control females); IDM: impact of disease in males (MS males vs control males); SDID: sex differential impact of disease ((MS females vs control females) - (MS males vs control males)). Protein-protein interaction network of genes increased in males (B, green) and females (C, orange) from those significant in all three comparisons. Edge thickness is directly proportional to the structural and functional confidence of the interaction. PPI: protein-protein interaction. Due to the high number of significant results, subclusters were calculated with the following specifications: 1) community search with the fastgreedy algorithm, 2) discarding of subclusters with a size smaller than 0.7, 3) disable text mining option and 4) genes set with a absolute magnitude of change higher of 4. (D) Degree of similarity between sex-differential enriched functions in females (left horizontal orange bars) and males (left horizontal green bars). Each row and column corresponds with a significant function. Blue intensity indicates the degree of similarity. Clusters are shown at the bottom, with the associated word cloud on the right of the plot.

#### **Figure S10. Oligodendrocytes sex-differential atlas in secondary progressive multiple sclerosis.** (A) Volcano plots of differential gene expression results. Each point represents a gene distributed by its magnitude of change (logFC, X-axis) and its significance (-log10(p.fitted value), X-axis) for each comparison. IDF: impact of disease in females (MS females vs control females); IDM: impact of disease in males (MS males vs control males); SDID: sex differential impact of disease ((MS females vs control females) - (MS males vs control males)). Protein-protein interaction network of genes increased in females (B, orange) and males (C, green) from those significant in all three comparisons. Edge thickness is directly proportional to the structural and functional confidence of the interaction. PPI: protein-protein interaction. (D) Degree of similarity between sex-differential enriched functions in females (left horizontal orange bars) and males (left horizontal green bars). Each row and column corresponds with a significant function. Blue intensity indicates the degree of similarity. Clusters are shown at the bottom, with the associated word cloud on the right of the plot.

#### **Figure S11. Oligodendrocyte precursor cells sex-differential atlas in secondary progressive multiple sclerosis.** (A) Volcano plots of differential gene expression results. Each point represents a gene distributed by its magnitude of change (logFC, X-axis) and its significance (-log10(p.fitted value), X-axis) for each comparison. IDF: impact of disease in females (MS females vs control females); IDM: impact of disease in males (MS males vs control males); SDID: sex differential impact of disease ((MS females vs control females) - (MS males vs control males)). Protein-protein interaction network of genes increased in females (B, orange) and males (C, green) from those significant in all three comparisons. Edge thickness is directly proportional to the structural and functional confidence of the interaction. PPI: protein-protein interaction. (D) Degree of similarity between sex-differential enriched functions in females (left horizontal orange bars) and males (left horizontal green bars). Each row and column corresponds with a significant function. Blue intensity indicates the degree of similarity. Clusters are shown at the bottom, with the associated word cloud on the right of the plot.

**Figure S12. Intersection of significant results among brain cell types for secondary progressive multiple sclerosis.** Upset plots for significant sex-differential (A) genes, (C) functions and (C) effectors of signaling pathways in the three assessed comparisons: IDF: impact of disease in females (MS females vs control females); IDM: impact of disease in males (MS males vs control males); SDID: sex differential impact of disease ((MS females vs control females) - (MS males vs control males)). OPCs: oligodendrocyte precursor cells.

**Figure S13. Significant results in the analyses of (A) signaling pathway effector activation and (B) cell-cell communication for the secondary progressive form of multiple sclerosis.** (A) Relative frequency distribution of KEGG signaling pathways significantly activated in females (orange) and males (green) by cell type. Individual terms have been classified into general categories (see legend). No significant results have been obtained for microglia and oligodendrocyte precursor cells. Oligodendrocytes presented more activated the *Tight junction* pathway in males. (D) Number of significant cell-cell interactions by group. Colour indicates the cell type providing the ligand protein. The thickness of the interaction corresponds to the magnitude of interactions. OPCs: oligodendrocyte precursor cells; KEGG: Kyoto Encyclopedia of Genes and Genomes.

**Figure S14. Upset plot depicting the overlap of sex-differentially expressed synaptic genes across neuronal subtypes.** Intersection of genes with sex-differential significant expression identified in all neurons, excitatory neurons, and inhibitory neurons (increased in females and increased in males, respectively). Set sizes are indicated by the horizontal bars on the left. Gene lists for each intersection are detailed in the colored boxes.

**Figure S15. Sex-differential patterns in glutamatergic and GABAergic signaling pathways in the validation dataset.** (A) Comparison of the sex differential impact of disease (SDID comparison) results between the UCSC-MS (all neurons, excitatory neurons and inhibitory neurons) and Macnair datasets. (B) Glutamatergic (top) and GABAergic (bottom) synaptic signaling pathways from analyzing Macnair *et al.* dataset. Nodes represent proteins, and edges represent interactions between them. Effector proteins correspond to the final node of each subpathway (arrowhead), pointing to the biological functions they regulate. *GABA: Gamma-aminobutyric acid.*

#### **Figure S16. Sex differential signaling pathways of (A) cholinergic, (B) serotonergic and (C) dopaminergic synapses in neurons from secondary progressive multiple sclerosis.** Nodes represent proteins of the signalling pathway and edges the interactions between nodes. Effector proteins are the last node in each subpathway (arrow point). The effector nodes point to the biological functions they exerted. GO: Gene Ontology; GABA: Gamma-aminobutyric acid. Orange edges: subpathways with increased activation in females; green edges: subpathways with increased activation in males.

#### **Figure S17. Significant functions in oligodendrocytes and oligodendrocyte precursor cells (OPCs) in secondary progressive multiple sclerosis.** Heatmap of oligodendrocytes and oligodendrocyte precursor cells by sex (columns) from the corresponding enriched functions (rows). Orange: functions enriched in females; green: functions enriched in males; grey: non significant enriched function.

#### **Figure S18. Summary of significant sex differences in relapsing-remitting multiple sclerosis.** (A) Expression pattern of marker genes (Y-axis) in B cells, CD4+ T cells, CD8+ T cells, NK cells, monocytes and dendritic cells (Y-axis). Each dot represents a cell, coloured according to the cell type noted. Marker genes have been selected from bibliographic research. (B) Number of cells identified for each cell type (X-axis) based on condition (Y-axis) and sex (color). MS: multiple sclerosis. (C) Number of significant features by cell type, analysis and comparison tested. (D) Upset map of significant features for each cell type separated by comparison and direction of change (logFC). Dots display whether the feature is significant, with specific colors indicating the comparison and logFC sign. Each row of numbers indicates, for each cell type and specific analysis, the number of significant features corresponding to the evaluated column in the dot map. Coloured squares highlight the significant features in the three comparisons IDF, IDM and SDID. (E) Number of significant cell-cell interactions by group. Colour indicates the cell type providing the ligand protein. The thickness of the interaction corresponds to the magnitude of interactions. IDF: impact of disease in females (MS females vs control females); IDM: impact of disease in males (MS males vs control males); SDID: sex differential impact of disease ((MS females vs control females) - (MS males vs control males)).

**Figure S19. Associations between number of cells and number of differentially expressed genes across cell types and comparisons for relapsing-remitting multiple sclerosis dataset.** Each point represents a specific cell type and comparison, colored by comparison group and shaped by cell type. The x-axis indicates the number of cells per condition, while the y-axis shows the number of significant differentially expressed genes (FDR < 0.05 and |logFC| > 0.5). Linear regression lines with 95% confidence intervals are shown in grey. Spearman correlation coefficients (rho) are indicated for each analysis.

#### **Figure S20. CD4+ T cells sex-differential atlas in relapsing-remitting multiple sclerosis.** (A) Volcano plots of differential gene expression results. Each point represents a gene distributed by its magnitude of change (logFC, X-axis) and its significance (-log10(p.fitted value), X-axis) for each comparison. IDF: impact of disease in females (MS females vs control females); IDM: impact of disease in males (MS males vs control males); SDID: sex differential impact of disease ((MS females vs control females) - (MS males vs control males)). Protein-protein interaction network of genes increased in females (B, orange) and males (C, green) from those significant in all three comparisons. Edge thickness is directly proportional to the structural and functional confidence of the interaction. PPI: protein-protein interaction. (D) Degree of similarity between sex-differential enriched functions in females (left horizontal orange bars) and males (left horizontal green bars). Each row and column corresponds with a significant function. Blue intensity indicates the degree of similarity. Clusters are shown at the bottom, with the associated word cloud on the right of the plot.

#### **Figure S21. CD8+ T cells sex-differential atlas in relapsing-remitting multiple sclerosis.** (A) Volcano plots of differential gene expression results. Each point represents a gene distributed by its magnitude of change (logFC, X-axis) and its significance (-log10(p.fitted value), X-axis) for each comparison. IDF: impact of disease in females (MS females vs control females); IDM: impact of disease in males (MS males vs control males); SDID: sex differential impact of disease ((MS females vs control females) - (MS males vs control males)). Protein-protein interaction network of genes increased in females (B, orange) and males (C, green) from those significant in all three comparisons. Edge thickness is directly proportional to the structural and functional confidence of the interaction. PPI: protein-protein interaction. (D) Degree of similarity between sex-differential enriched functions in females (left horizontal orange bars) and males (left horizontal green bars). Each row and column corresponds with a significant function. Blue intensity indicates the degree of similarity. Clusters are shown at the bottom, with the associated word cloud on the right of the plot.

**Figure S22. NK cells sex-differential atlas in relapsing-remitting multiple sclerosis.** (A) Volcano plots of differential gene expression results. Each point represents a gene distributed by its magnitude of change (logFC, X-axis) and its significance (-log10(p.fitted value), X-axis) for each comparison. IDF: impact of disease in females (MS females vs control females); IDM: impact of disease in males (MS males vs control males); SDID: sex differential impact of disease ((MS females vs control females) - (MS males vs control males)). Protein-protein interaction network of genes increased in females (B) from those significant in all three comparisons. Edge thickness is directly proportional to the structural and functional confidence of the interaction. PPI: protein-protein interaction. Males only have increased CCL4 and HBA2 genes. (C) Degree of similarity between sex-differential enriched functions in females (left horizontal orange bars) and males (left horizontal green bars). Each row and column corresponds with a significant function. Blue intensity indicates the degree of similarity. Clusters are shown at the bottom, with the associated word cloud on the right of the plot. NK: natural killer.

#### **Figure S23. Monocytes sex-differential atlas in relapsing-remitting multiple sclerosis.** (A) Volcano plots of differential gene expression results. Each point represents a gene distributed by its magnitude of change (logFC, X-axis) and its significance (-log10(p.fitted value), X-axis) for each comparison. IDF: impact of disease in females (MS females vs control females); IDM: impact of disease in males (MS males vs control males); SDID: sex differential impact of disease ((MS females vs control females) - (MS males vs control males)). Protein-protein interaction network of genes increased in females (B, orange) and males (C, green) from those significant in all three comparisons. Edge thickness is directly proportional to the structural and functional confidence of the interaction. PPI: protein-protein interaction. (D) Degree of similarity between sex-differential enriched functions in females (left horizontal orange bars) and males (left horizontal green bars). Each row and column corresponds with a significant function. Blue intensity indicates the degree of similarity. Clusters are shown at the bottom, with the associated word cloud on the right of the plot.

#### **Figure S24. (A) B cells and (B) dendritic cells sex-differential atlas in relapsing-remitting multiple sclerosis.** Volcano plots of differential gene expression results. Each point represents a gene distributed by its magnitude of change (logFC, X-axis) and its significance (-log10(p.fitted value), X-axis) for each comparison. IDF: impact of disease in females (MS females vs control females); IDM: impact of disease in males (MS males vs control males); SDID: sex differential impact of disease ((MS females vs control females) - (MS males vs control males)).

#### **Figure S25. Detailed sex-differential significant changes in the activation of signaling pathways and interaction strengths in cell-cell communication for relapsing-remitting multiple sclerosis.** (A) Effectors with increased activation in females (orange boxes) and males (green boxes). Each column represents a different cell type, and each row corresponds to a signaling pathway. Within each pathway, significant effectors for at least one cell type are highlighted. The numeric value at the end of each row denotes the count of subpathways within the pathway that lack significance in any cell type. (B-C) Relative (right) and absolute (left) information flow of pathways in females (B) and males (C) when comparing multiple sclerosis to control samples. Cell types evaluated: CD4+ T cells, CD8+ T cells, monocytes and NK cells. Information flow was calculated as the sum of communication probability (i.e. interaction strengths) among all ligand-receptor pairs in each group. Paired Wilcoxon test was implemented to determine significant differences, which are highlighted by coloring their names.

**Figure S26. Upset plot for genes more expressed in males than females by cell type in relapsing-remitting multiple sclerosis.** Horizontal bars represent the number of significant genes in each cell type. Dots indicate the combinations of intersections tested, with the top vertical bars denoting the number of significant genes of the corresponding intersection.

#### **Figure S27. Summary of significant sex differences in primary progressive multiple sclerosis.** (A) Expression pattern of marker genes (Y-axis) in B cells, CD4+ T cells, CD8+ T cells, NK cells, monocytes and dendritic cells (Y-axis). Each dot represents a cell, coloured according to the cell type noted. Marker genes have been selected from bibliographic research. (B) Number of cells identified for each cell type (X-axis) based on condition (Y-axis) and sex (colour). MS: multiple sclerosis. (C) Number of significant features by cell type, analysis and comparison tested. (D) Upset map of significant features for each cell type separated by comparison and direction of change (logFC). Dots display whether the feature is significant, with specific colours indicating the comparison and logFC sign. Each row of numbers indicates, for each cell type and specific analysis, the number of significant features corresponding to the evaluated column in the dot map. Coloured squares highlight the significant features in the three comparisons IDF, IDM and SDID. (E) Number of significant cell-cell interactions by group. Colour indicates the cell type providing the ligand protein. The thickness of the interaction corresponds to the magnitude of interactions. IDF: impact of disease in females (MS females vs control females); IDM: impact of disease in males (MS males vs control males); SDID: sex differential impact of disease ((MS females vs control females) - (MS males vs control males)). NK: natural killer.

**Figure S28. Associations between number of cells and number of differentially expressed genes across cell types and comparisons for primary progressive multiple sclerosis dataset.** Each point represents a specific cell type and comparison, colored by comparison group and shaped by cell type. The x-axis indicates the number of cells per condition, while the y-axis shows the number of significant differentially expressed genes (FDR < 0.05 and |logFC| > 0.5). Linear regression lines with 95% confidence intervals are shown in grey. Spearman correlation coefficients (rho) are indicated for each analysis.

#### **Figure S29. CD4+ T cells sex-differential atlas in primary progressive multiple sclerosis.** (A) Volcano plots of differential gene expression results. Each point represents a gene distributed by its magnitude of change (logFC, X-axis) and its significance (-log10(p.fitted value), X-axis) for each comparison. IDF: impact of disease in females (MS females vs control females); IDM: impact of disease in males (MS males vs control males); SDID: sex differential impact of disease ((MS females vs control females) - (MS males vs control males)). Protein-protein interaction network of genes increased in females (B, orange) and males (C, green) from those significant in all three comparisons. Edge thickness is directly proportional to the structural and functional confidence of the interaction. PPI: protein-protein interaction. (D) Degree of similarity between sex-differential enriched functions in females (left horizontal orange bars) and males (left horizontal green bars). Each row and column corresponds with a significant function. Blue intensity indicates the degree of similarity. Clusters are shown at the bottom, with the associated word cloud on the right of the plot.

#### **Figure S30. CD8+ T cells sex-differential atlas in primary progressive multiple sclerosis.** (A) Volcano plots of differential gene expression results. Each point represents a gene distributed by its magnitude of change (logFC, X-axis) and its significance (-log10(p.fitted value), X-axis) for each comparison. IDF: impact of disease in females (MS females vs control females); IDM: impact of disease in males (MS males vs control males); SDID: sex differential impact of disease ((MS females vs control females) - (MS males vs control males)). Protein-protein interaction network of genes increased in females (B, orange) and males (C, green) from those significant in all three comparisons. Edge thickness is directly proportional to the structural and functional confidence of the interaction. PPI: protein-protein interaction. (D) Degree of similarity between sex-differential enriched functions in females (left horizontal orange bars) and males (left horizontal green bars). Each row and column corresponds with a significant function. Blue intensity indicates the degree of similarity. Clusters are shown at the bottom, with the associated word cloud on the right of the plot.

#### **Figure S31. NK cells sex-differential atlas in primary progressive multiple sclerosis.** (A) Volcano plots of differential gene expression results. Each point represents a gene distributed by its magnitude of change (logFC, X-axis) and its significance (-log10(p.fitted value), X-axis) for each comparison. IDF: impact of disease in females (MS females vs control females); IDM: impact of disease in males (MS males vs control males); SDID: sex differential impact of disease ((MS females vs control females) - (MS males vs control males)). Protein-protein interaction network of genes increased in females (B, orange) and males (C, green) from those significant in all three comparisons. Edge thickness is directly proportional to the structural and functional confidence of the interaction. PPI: protein-protein interaction. (D) Degree of similarity between sex-differential enriched functions in females (left horizontal orange bars) and males (left horizontal green bars). Each row and column corresponds with a significant function. Blue intensity indicates the degree of similarity. Clusters are shown at the bottom, with the associated word cloud on the right of the plot.

#### **Figure S32. Monocytes sex-differential atlas in primary progressive multiple sclerosis.** (A) Volcano plots of differential gene expression results. Each point represents a gene distributed by its magnitude of change (logFC, X-axis) and its significance (-log10(p.fitted value), X-axis) for each comparison. IDF: impact of disease in females (MS females vs control females); IDM: impact of disease in males (MS males vs control males); SDID: sex differential impact of disease ((MS females vs control females) - (MS males vs control males)). Protein-protein interaction network of genes increased in females (B, orange) and males (C, green) from those significant in all three comparisons. Edge thickness is directly proportional to the structural and functional confidence of the interaction. PPI: protein-protein interaction. (D) Degree of similarity between sex-differential enriched functions in females (left horizontal orange bars) and males (left horizontal green bars). Each row and column corresponds with a significant function. Blue intensity indicates the degree of similarity. Clusters are shown at the bottom, with the associated word cloud on the right of the plot.

#### **Figure S33. (A, C) B cells and (B, D) dendritic cells sex-differential atlas in primary progressive multiple sclerosis.** (A,B) Volcano plots of differential gene expression results. Each point represents a gene distributed by its magnitude of change (logFC, X-axis) and its significance (-log10(p.fitted value), X-axis) for each comparison. IDF: impact of disease in females (MS females vs control females); IDM: impact of disease in males (MS males vs control males); SDID: sex differential impact of disease ((MS females vs control females) - (MS males vs control males)). (C,D) Degree of similarity between sex-differential enriched functions in females (left horizontal orange bars) and males (left horizontal green bars). Each row and column corresponds with a significant function. Blue intensity indicates the degree of similarity. Clusters are shown at the bottom, with the associated word cloud on the right of the plot.

#### **Figure S34. Detailed sex-differential significant changes in the activation of signaling pathways for primary progressive multiple sclerosis.** Effectors with increased activation in females (orange boxes) and males (green boxes). Each column represents a different cell type, and each row corresponds to a signaling pathway. Within each pathway, significant effectors for at least one cell type are highlighted. The numeric value at the end of each row denotes the count of subpathways within the pathway that lack significance in any cell type.

#### **Figure S35. Detailed sex-differential significant changes in the cell-cell communication interaction strengths for primary progressive multiple sclerosis.** Relative (right) and absolute (left) information flow of pathways in females (A) and males (B) when comparing multiple sclerosis to control samples. Cell types evaluated: CD4+ T cells, CD8+ T cells, monocytes and NK cells. Information flow was calculated as the sum of communication probability (i.e. interaction strengths) among all ligand-receptor pairs in each group. Paired Wilcoxon test was implemented to determine significant differences, which are highlighted by coloring their names. NK: natural killer.

**Figure S36. Protein-protein interaction network of the genes that provide clustering of RRMS and PPMS immune system cell types according to their differential sex profile.** Network showing the connection of CD4+ T cells, CD8+ T cells, NK cells and monocytes in RRMS and PPMS subtypes from genes with significant expression by sex in at least three of the eight genes evaluated. Edge thickness indicates the structural and functional confidence of the interaction. Colour established based on the functions the genes are involved in. RRMS: relapsing-remitting multiple sclerosis; PPMS: primary progressive multiple sclerosis; NK: natural killer.

#### **Figure S37. Cell distribution by sample (donor).** Each dot represents a cell, coloured by donor. (A) UCSC-MS dataset, (B) GSE144744 cohort 1 and (C) GSE144744 cohort 3.
